# MONISEDA Project: Improving Analgosedation Monitoring in Spanish Pediatric Intensive Care Units

**DOI:** 10.3389/fped.2021.781509

**Published:** 2021-12-07

**Authors:** Santiago Mencía, Raquel Cieza, Jimena del Castillo, Jesús López-Herce, Sedation Group of Spanish Pediatric Critical Care Society (SECIP), Rocío Tapia

**Affiliations:** ^1^Pediatric Intensive Care Department, Gregorio Marañón General University Hospital, Madrid, Spain; ^2^Public Health and Mother-Child Department, School of Medicine, Complutense University, Madrid, Spain; ^3^Gregorio Marañón Health Research Institute, Madrid, Spain; ^4^Mother-Child Health and Development Network (Red SAMID) of ISCIII-Sub-Directorate General for Research Assessment and Promotion, European Regional Development Fund, Madrid, Spain

**Keywords:** monitoring analgosedation, MONISEDA project, PICU, withdrawal scales, delirium scales

## Abstract

**Background:** Analgosedation (AS) assessment using clinical scales is crucial to follow the international recommendations about analgosedation. The Analgosedation workgroup of the Spanish Society of Pediatric Intensive Care (SECIP) carried out two surveys in 2008 and 2015, which verified the gap in analgosedation assessment in Spanish pediatric intensive care unit (PICUs). The objective of the study was to analyze how analgosedation assessment by clinical scales changed after a multicenter intervention program.

**Methods:** This is a multicenter pre–post study comparing the use of sedation, analgesia, withdrawal, and delirium scales before and after the MONISEDA project. Results were also compared with a control group formed by non-participating units. A survey about analgosedation management and monitoring was filled out before (year 2015) and after (year 2020) the implementation of the MONISEDA project in 2016. Results were compared not only between those periods of time but also between participant and non-participant PICUs in the MONISEDA project (M-group and non-M group, respectively). Data related to analgosedation of all patients admitted to a MONISEDA-participant PICU were also collected for 2 months.

**Results:** Fifteen Spanish PICUs were enrolled in the MONISEDA project and another 15 non-participant PICUs formed the control group. In the M-group, the number of PICUs with a written analgosedation protocol increased from 53 to 100% (*p* = 0.003) and withdrawal protocol from 53 to 100% (*p* = 0.003), whereas in the non-M group, the written AS protocol increased from 80 to 87% and the withdrawal protocol stayed on 80%. The number of PICUs with an analgosedation team increased from 7 to 47% in the M-group (*p* = 0.01) and from 13 to 33% in the non-M group (*p* = 0.25). In the M-group, routine use of analgosedation clinical scales increased from 7 to 100% (*p* < 0.001), withdrawal scales from 7% to 86% (*p* = 0.001), and delirium scales from 7 to 33% (*p* = 0.125). In the non-M group, the number of PICUs using AS scales increased from 13 to 100% (*p* < 0.001), withdrawal scales from 7 to 27% (*p* = 0.125), and delirium scales from 0 to 7% (*p* = 1).

**Conclusions:** The development of a specific training program improves monitoring and management of analgosedation in PICUs.

## Introduction

Sedation and analgesia are essential in the management of critically ill children. Article 24 of the United Nations Convention on the Rights of Children addresses the rights and special demands of children in healthcare institutions, recognizing that children are especially vulnerable. It defines the right to enjoy the highest attainable standard of health and the right to avoid pain, fear, and stress.

That is why pain and anxiety abolition in children must be a priority. An adequate analgosedation diminishes emotional stress, facilitates nursing care, allows adaptation to mechanical ventilation, and improves prognosis, reducing the length of mechanical ventilation and pediatric intensive care unit (PICU) stay (1, 2). However, sedative and analgesic drugs can cause adverse effects and increase morbidity and mortality (3).

International recommendations highlight the importance of improving comfortability in critically ill children, mainly through proper analgesia, minimal possible sedation, and measures to prevent withdrawal syndrome and delirium (4, 5). For that purpose, it is necessary to assess and treat pain prior to administration of sedatives and to keep minimal sedation to allow patients to interact with the environment without agitation. It is fundamental to apply valid and reliable assessment tools to identify pain, excessive or insufficient sedation, and delirium in critically ill children and to use them on a routine basis, adjusting our procedures according to its rating (6–9).

The Analgosedation workgroup of the Spanish Society of Pediatric Intensive Care (SECIP) carried out two surveys in 2008 and 2015, which verified the gap in analgosedation assessment in Spanish PICUs. Therefore, the group decided to perform a training program called MONISEDA project. Its objectives were to create analgosedation working teams in each Spanish PICU and to promote and unify analgosedation clinical scales to improve the assessment of pain, stress, iatrogenic withdrawal syndrome, and delirium.

## Materials and Methods

A multicenter pre–post study comparing the use of sedation, analgesia, withdrawal, and delirium scales before and after the MONISEDA project was performed. The project was advertised on the SECIP website, and all the Spanish PICUs that were interested had the opportunity to participate in it. The Institutional Review Board reviewed the study and approved it (EPA-SP 02/2017), and written informed consent was waived. This manuscript adheres to the applicable STROBE guidelines.

The MONISEDA project was divided into different stages:

1. *Preliminary survey:* A survey was performed in 2015 to determine how pain and stress were being managed and monitored in PICUs at that moment. Two groups were included: PICUs participating in the MONISEDA project (M-group) and non-participants (non-M group).
2. *Development of MONISEDA training program* (Table 1): Informative and training activities with sessions and workshops for all PICU members were conducted for a period of 2 months. The project encouraged the creation of an analgosedation team in each PICU of the M-group, consisting of one or two doctors and four–six nurses.

**Table 1:** Phases of the MONISEDA project training in each PICU.

**Periods**	**Duration**	**Description**
Analgosedation teams	15 days	- Creation of a team in each PICU that will be composed of 1 or 2 physicians and 4 to 6 PICU nurses. - Implementation or reinforcement of scales and analgosedation protocol.
Information period	15 days	- Presentation of the project to the rest of the PICU staff members via: • General session. • Workshops regarding clinical scales of analgesia, sedation, IWS and delirium (driven by Analgosedation team nurses).
Training period	1 month	- All nurses will assess and register analgesia, sedation, iatrogenic withdrawal syndrome and delirium using scales once per shift. - All doubts will be discussed with the Analgosedation team members.
Data collection period	2 months	- Data collection from each patient concerning analgosedation by specific team members. - Data analysis and evaluation of results.

*IWS, iatrogenic withdrawal syndrome*.

The analgosedation working team was responsible for the training of the rest of the PICU staff through an informative clinical session and a practical training workshop on the use of clinical scales and data collection specially addressed to the nurses of the unit. Once the personnel had been formed, a training period of 1 month was carried out, during which the same data were collected as in the study. During this period, any doubts that arose during the application of the different scales were resolved.

3. *Data collection phase:* For 2 months, PICUs participating in MONISEDA project filled out the data collection form. After obtaining informed consent from parents or guardians, the scores for the analgesia clinical scale (adapted to age) and sedation (COMFORT scale) for all children admitted to the PICU were registered once per shift (6 a.m., 2 p.m., and 9 p.m.). No patients were excluded. Data was sent to the coordinator center for its analysis. All study coordinators from the different PICUs were asked to complete a satisfaction survey upon completion of this phase.
4. *Subsequent survey after the project:* In 2020, the same data collection form was again completed by all the PICUS of both groups, in order to compare these results with the previous ones.

### Statistical Analysis

All data was analyzed by the software package SPSS for Windows, version 19. Qualitative variables were expressed as percentages, and quantitative variables as means and standard deviation. Fisher's exact test was used to compare qualitative variables, and Mann–Whitney *U*-test for quantitative variables. The McNemar test for related samples was used to analyze the evolution of the variables of the 2020 survey with respect to that of 2015. Statistical significance was considered when *p* < 0.05.

## Results

### Analgosedation Survey in 2015

Table 2 shows the results of the first analgosedation survey, comparing PICUs of the M-group and the non-M group. The number of PICUs that followed a written analgosedation and withdrawal protocol was higher in the non-M group, although differences were not statistically significant.

**Table 2:** Initial analgosedation survey (year 2015).

**Variable**	**Global results** **(%)**	**Moniseda (%)**	**Non-Moniseda (%)**	***P***
Written AS protocol	67	53	80	0.123
Use of daily AS scales	10	7	13	0.5
AS working team creation	10	7	13	0.5
Sedation scale used	71	Ramsay	Ramsay	0.5
		76	66	
Objective monitoring: BIS	30	33	27	0.5
Written WS protocol	67	53	80	0.123
Use of daily IWS scales	3	7	0	0.5
Usual use of delirium scales	0	7	0	–

*Comparison between MONISEDA group and non-MONISEDA group*.

*AS, analgosedation; BIS, bispectral index; IWS, iatrogenic withdrawal syndrome*.

### MONISEDA Project 2016

In the M-group, the project was introduced to the rest of the staff in 85% of the PICUs, and a specific analgosedation working team consisting of doctors and nurses was created in 61% of the units.

At the end of the project, a satisfaction survey was completed by the coordinators of each PICU (Table 3). Main difficulties to implement monitoring were the lack of habit and the workload, principally from the nurses' point of view (40%). There were 33% of doctors and 31% of nurses who thought that the project had significantly changed routine analgosedation monitoring, and 70% of participants considered that some monitoring aspects had changed. The most important improvement was the incorporation of the use of clinical monitoring scales in 33% of the units. A greater use of sedation scales was attained in 10 PICUs, of withdrawal scales in six units, and of analgesia scales in three units. Four PICUs started to use delirium scales.

**Table 3:** Satisfaction survey of MONISEDA group 2016.

**Variable**	**MONISEDA Project (%)**
AS working team creation	61.5
Difficulties to develop the project	40
Changes in AS daily management	69
Daily AS monitoring implementation	33
Daily IWS monitoring implementation	40

*AS, analgosedation; IWS, iatrogenic withdrawal syndrome*.

During the 2 months of the study, data from 489 children were collected [55% were males, mean age was 4.2 years old (SD 4.7), and mean weight was 21 kg (SD 18)]. The reason for admission was medical pathologies in 53% of the cases. The mean length of stay was 6.3 days (SD 13); 30% of the patients underwent mechanical ventilation and 1.8% of the patients died.

Analgesia was monitored by scales in 97% of the patients, with a mean score of 1.5 (SD 1.4). Sedation assessment was performed by the COMFORT scale in 93% of the patients, with an average rating of 18.3 (SD 5.9). Bispectral index (BIS) monitoring of the level of consciousness was used in 8% of the patients, with a mean score of 56 (SD 14).

### Analgosedation Survey in 2020

Table 4 shows the results of the last analgosedation survey performed, comparing PICUs of the M-group and the non-M group. Every PICU (100%) in both groups used some analgosedation clinical scale on a daily basis. PICUs in the M-group performed analgosedation assessment more frequently than the control group. Statistically significant differences were found for withdrawal monitoring (87 vs. 27%; *p* = 0.001). Delirium assessment increased importantly too (33 vs. 7%; *p* = 0.08) but did not reach statistical significance.

**Table 4:** Final analgosedation survey (year 2020).

**Variable**	**Global results** **(%)**	**Moniseda** **(%)**	**Non-Moniseda (%)**	***P***
Written AS protocol	93	100	87	0.5
Use of daily AS scales	100	100	100	–
AS working team creation	40	47	33	0.355
Sedation scale used		COMFORT	COMFORT	
		100	66	
Objective monitoring: BIS	60	60	60	0.645
Written IWS protocol	90	100	80	0.241
Use of daily WS scales	57	87	27	**0.001**
Usual use of delirium scales	20	33	7	0.080

*Comparison between MONISEDA group and non-MONISEDA group*.

*AS, analgosedation; BIS, biespectral index; IWS, iatrogenic withdrawal syndrome. The bold values mean the percentage increase in the different variables in both groups*.

### Comparison Between 2015 and 2020 Surveys

**Figure 1:**
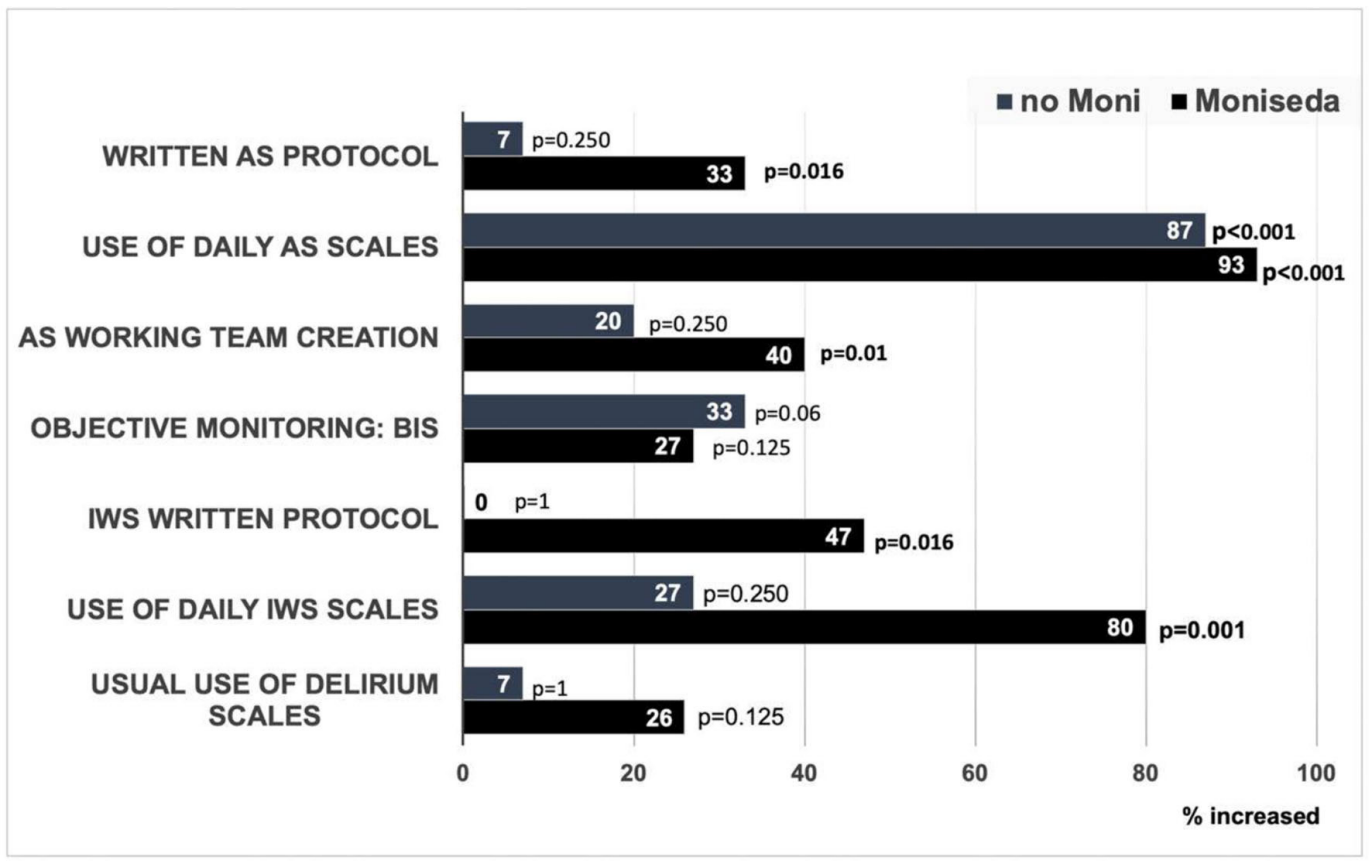
Improvement in analgosedation survey 2020 compared to 2015 in both the MONISEDA and non-MONISEDA groups. AS, analgosedation; BIS, bispectral index, IWS, iatrogenic withdrawal syndrome.

Figure 1 shows the comparison between the first and the last analgosedation survey. In both groups, the use of clinical scales improved. In recent years, important morbidities have been described in patients admitted to intensive care units related to the inappropriate use of analgosedation. The scientific community has improved awareness of this problem. For this reason, most hospitals have optimized the use of analgesia, which requires adequate monitoring by means of validated scales.

The increase of analgosedation monitoring activities was higher in the M-group than in the non-M group. There was a higher increment in the creation of analgosedation working teams and written protocols for withdrawal monitoring.

## Discussion

This is the first multicenter project that aims to improve analgosedation monitoring in Spanish PICUs. Our study shows that a specific training project significantly improves analgesia, sedation, and withdrawal monitoring. It enhances awareness of health professionals and facilitates the creation of analgosedation working teams consisting of doctors and nurses. The patients admitted to PICU are complex patients with a high care load. The performance and the recording of the analgosedation and delirium scales can lead to an overload of work for the nursing staff. In addition, the implementation of the new work routines sometimes generates rejection, mainly related to the lack of knowledge about them. With an adequate training on their application and their importance, both points can be improved.

This leads to regular and long-term monitoring after the educational intervention. Our project could be a model for the development of new similar projects in other countries.

In the second survey, conducted 5 years after the intervention, it is important to highlight the improvement in the daily monitoring of analgosedation, withdrawal, and delirium. Creation of multidisciplinary working teams (doctors and nurses) and a better follow-up of the recommendations to homogenize the use of clinical scales in the Spanish PICUs have significantly increased too. Furthermore, the use of the COMFORT/COMFORT-b scale (specific for pediatric patients) raised compared to the Ramsay scale, which is only validated for adults (10, 11).

The analysis showed that over the 2 months of data collection, analgosedation monitoring followed the international recommendations. The implementation of analgesia scales per shift achieved good pain control. Based on an early diagnosis and treatment adjustment according to the score, most of the patients showed no pain or mild pain and an appropriate level of sedation (4).

The second survey, conducted 5 years after the intervention, showed an improvement of analgosedation monitoring in both the M and non-M groups, which reflected a growing awareness of PICU health professionals on the importance of this monitoring (12, 13). We think that there could have been a contagion or spread effect from the PICUs included in the MONISEDA project to the rest of Spanish PICUs (14, 15).

Other studies have previously highlighted the importance of an appropriate analgosedation (AS) monitoring in order to prevent and manage the appearance of withdrawal syndrome or delirium (16–18).

Achieving an improvement on AS and withdrawal monitoring in the Spanish PICUs is challenging. The implementation of a new routine in a clinical service is difficult, especially when it is a highly complex unit and there is a high staff turnover. Both time and a great effort are essential to accomplish this task. Difficulties to introduce these types of protocols are mentioned in other studies (19, 20). We consider that the creation of working teams made up of doctors and nurses is very important. The engagement and training of the nursing staff are crucial as they are in charge of the AS monitoring and the adjustment of the treatment to the patient's condition (16). However, despite the observed improvement in the 2020 survey, there are still some aspects, such as the withdrawal and delirium monitoring (16, 21, 22), which need to be enhanced and require continuous evaluation and feedback.

Our study has some limitations. Despite every Spanish PICU being invited to participate in the project, only one third of them accepted the invitation. So, probably, those PICUs included in the project were also those with higher awareness on the importance of analgosedation and those that felt the need to implement these protocols. The hospitals that did not participate in the study did complete an online survey recording the management of analgosedation in their work units. This concern could have introduced a bias in the comparison of both groups and could explain the fact that in the initial survey only a small percentage of PICUs followed a written AS protocol.

Another limitation is that the observational study of patients was not repeated in 2020 to verify the improvements observed in the survey in the daily practice.

The study could be improved by having a longer duration. In this way, the training of the team and the different stages of the study could be repeated periodically, comparing the results after several training stages.

In conclusion, we think that the creation of multicenter training projects, like the MONISEDA project, could be an effective tool to achieve a better analgosedation assessment of critically ill children. Our project could serve as a model for other countries, adjusting it to their specific characteristics.

## Data Availability Statement

The original contributions presented in the study are included in the article/supplementary material, further inquiries can be directed to the corresponding author/s.

## Ethics Statement

The studies involving human participants were reviewed and approved by the Institutional Review Board (EPA-SP 02/2017). Written informed consent to participate in this study was provided by the participants' legal guardian/next of kin.

## Sedation Group of Spanish Pediatric Critical Care Society (SECIP)

Rocío Tapia, Ramón y Cajal Hospital, Madrid; Raúl Borrego, Virgen de la Salud Hospital, Toledo; Mercedes Domínguez, Miguel Servet Hospital, Zaragoza; Cristina Calvo, Donostia University Hospital, Donostia; Francisco Fernández, Salamanca University Hospital, Salamanca; Manuel Nieto, Cruces Hospital, Bilbao; Ana Estalella, Puerta del Mar Hospital, Cádiz; Ana Vivanco, Príncipe de Asturias Hospital, Oviedo; José Fernández-Cantalejo, Fundación Jiménez Díaz Hospital, Madrid; David Lozano, La Mancha Centro Hospital, Ciudad Real; Esther Aleo, Clínico San Carlos Hospital, Madrid; Raúl Montero, Reina Sofía Hospital, Córdoba; Cristina Yun, Carlos Haya Hospital, Málaga; Artur Sharluyan, Son Espases Hospital, Palma de Mallorca; Mónica Riaza, Montepríncipe Hospital, Madrid; Alba Palacios, Doce de Octubre Hospital, Madrid; Antonio Rodríguez-Núñez, Clínico University, Santiago de Compostela.

## Author Contributions

SM helped in manuscript writing, study design, programming, data acquisition, and data validation. RC helped as methodology advisor and with manuscript editing. JC helped with manuscript editing. JL-H helped in study design and in manuscript writing. SECIP authors helped in data acquisition and data validation. All authors contributed to the article and approved the submitted version.

## Conflict of Interest

The authors declare that the research was conducted in the absence of any commercial or financial relationships that could be construed as a potential conflict of interest.

## Publisher's Note

All claims expressed in this article are solely those of the authors and do not necessarily represent those of their affiliated organizations, or those of the publisher, the editors and the reviewers. Any product that may be evaluated in this article, or claim that may be made by its manufacturer, is not guaranteed or endorsed by the publisher.

## Abbreviations

AS: analgosedation
SECIP: Spanish Society of Pediatric Intensive Care
IWS: iatrogenic withdrawal syndrome
BIS: bispectral index
PICU: pediatric intensive care unit.
